# From symbolism to substance: evaluating state disclosure laws and the case for federal oversight

**DOI:** 10.3389/frhs.2025.1666949

**Published:** 2025-09-24

**Authors:** Sherri Cheng, Wenjing Duan, Wenqi Zhou

**Affiliations:** ^1^ Department of Operation Management and Business Technology Management, Chaifetz School of Business, Saint Louis University, Saint Louis, MO, United States; ^2^ Information Systems & Technology Management, School of Business, The George Washington University, Washington, DC, United States; ^3^ Department of Information Systems and Technology, Palumbo-Donahue School of Business, Duquesne University, Pittsburgh, PA, United States

**Keywords:** difference in differences, open payment, payment disclosure regulation, PPSA, state disclosure law, transparency

## Abstract

Pharmaceutical payments to physicians have long raised concerns about conflicts of interest and rising healthcare costs. While the Physician Payments Sunshine Act (PPSA) established federal transparency standards in 2013, several states had already enacted their own disclosure laws. This study evaluates the hypothesis that individual state policies varied in effectiveness at shaping corporate payment strategies. Specifically, we examined four state policies, Massachusetts (MA), Maine (ME), Minnesota (MN), and West Virginia (WV), using a difference-in-differences design, comparing the outcomes with matched control states before and after PPSA implementation. We analyzed meals and travel payments from four major pharmaceutical companies using ProPublica's Dollars for Docs (2012–2013) and Centers for Medicare & Medicaid Services’ Open Payments (2014–2015) data. Results indicate that MA's comprehensive policy, featuring strict reporting requirements, public accessibility, and enforcement, yielded a significant 44% decline in travel payments. In contrast, ME, MN, and WV's policies showed negligible impacts. These findings suggest that disclosure laws exert real influence only when they are comprehensive, transparent, and backed by enforcement. This underscores the critical role of policy structure in addressing conflicts of interest and points to ways the PPSA framework could be strengthened. Based on our findings, we suggest that other nations could strengthen their systems by adopting centralized and standardized reporting systems, similar to the PPSA.

## 1 Introduction

Financial relationships between pharmaceutical companies and physicians have long fueled concerns in the United States (U.S.) about conflicts of interest and escalating healthcare costs (1–4). To address these concerns, several states implemented their own disclosure laws prior to the enactment of the federal Physician Payments Sunshine Act (PPSA) in August 2013 (4). The PPSA established a nationwide, standardized disclosure system, requiring pharmaceutical and medical device manufacturers to report payments or transfers of value to physicians and teaching hospitals. Payments are categorized into standardized groups, submitted centrally to Centers for Medicare & Medicaid Services (CMS), and made publicly accessible via the Open Payments website. Penalties are imposed for noncompliance, aiming to enhance transparency and uncover potential conflicts of interest. By comparison, state disclosure policies varied considerably in scope and enforcement.

Empirical studies of these early state laws report mixed outcomes. Pham-Kanter, Alexander, and Nair (5) found negligible effects on branded prescribing in Maine (ME) and West Virginia (WV), whereas King and Bearman (4) showed that gift bans, rather than disclosure alone, produced larger reductions in drug diffusion. Massachusetts' more stringent policy yielded variable outcomes: Guo et al. (1) documented declines in both branded and generic prescriptions, while Chao and Larkin (6) found reductions concentrated among heavy brand-name prescribers.

Much of the prior literature has treated state-level disclosure laws as largely uniform, grouping diverse policies under a single umbrella of “state regulations” [e.g., (7–9)]. This approach obscures meaningful differences in policy design, including reporting thresholds, data accessibility, and enforcement mechanisms, which could be critical in shaping corporate responses. For example, studies examining the impact of early disclosure laws often grouped Massachusetts (MA) with states such as Minnesota (MN) and WV, without distinguishing MA's stringent requirements—public databases, detailed reporting, and penalties—from the limited reach and weak enforcement in MN and WV (7, 9).

Our study departs from this uniform treatment by explicitly disaggregating the effects of individual state regimes. Using a difference-in-differences (DID) framework (10) with matched control states, we examine the effects of four state policies (MA, ME, MN, WV) on industry payments—total payments and number of physicians receiving payments—for meals and travel. We find that only MA's robust law significantly reduced discretionary payments, particularly for travel, whereas weaker or less transparent laws had little measurable effect. This differentiated perspective provides a clearer picture of the pre-PPSA policy landscape, highlighting that effectiveness depends not only on the presence of a disclosure law but also on the strength, scope, and enforceability of its provisions.

At the international level, countries such as Australia, Japan, and several in Europe have implemented transparency policies, though with notable differences from the U.S. PPSA in design and implementation. In Australia, Medicines Australia's Code requires member companies to disclose payments to healthcare professionals (HCPs), but reporting is limited to members and depends on HCP consent. In Japan, disclosure is self-regulated on company websites, without a centralized database. In Europe, France's Bertrand Law mandates HCP payment disclosure via the ANSM, while other countries (Portugal, Latvia, Greece, Denmark) vary in thresholds, coverage, and data accessibility.

By contrast, the PPSA in the U.S. offers a nationwide, standardized, fully public system with clear enforcement, providing greater consistency, accessibility, and accountability. Taken together, our findings highlight the importance of policy design in generating substantive behavioral change and underscore their relevance for refining both federal and international transparency frameworks.

## 2 Methods

The theoretical foundation for this study rests on the premise that transparency might influence industry behavior by reshaping incentives and reducing information asymmetries. Disclosure requirements make financial relationships between physicians and industry visible to regulators, patients, and the public, thereby increasing reputational and regulatory risks for non-compliant or excessive payments (11, 12). This visibility encourages firms to adopt more cautious marketing practices and limits opportunities for undue influence on prescribing patterns (13, 14). Thus, well-designed transparency policies can mitigate conflicts of interest by curbing distortive financial relationships (4, 7).

Building on this conceptual foundation, we empirically tested the extent to which state-level disclosure laws shaped industry payment behavior. Specifically, we evaluated the following hypothesis and then conducted a systematic comparison to assess how differences in the design and enforcement of individual states' disclosure policies help explain the observed effects.

**Hypothesis:** Individual state-level disclosure policies vary in their effectiveness at reducing pharmaceutical company payments to physicians, relative to the standardized, nationwide PPSA.

The nationwide implementation of the PPSA in August 2013 offers a quasi-experiment setup, where states experienced varying levels of payment disclosure regulation prior to 2013 and were subsequently subjected to a standardized federal mandate. To test this hypothesis, we constructed a dataset by combining payment data from ProPublica's Dollars for Docs (PDFD) for the period before the PPSA's enactment (2012–2013) and CMS Open Payments databases for the periods after the PPSA's enactment (2014–2015). Before the implementation of PPSA, five states, MA, ME, MN, Vermont (VT), and WV, along with Washington, D.C. (DC) had policies requiring pharmaceutical companies to disclose payments to healthcare professionals. However, because DC's unique characteristics made it difficult to identify a comparable match, and missing data for Eli Lilly and Novartis in multiple years limited the reliability of VT's records. Thus, we excluded both DC and VT from the DID analysis. We matched each of the remaining disclosure states (MA, ME, MN, WV) with a comparable treatment state (Maryland, Oklahoma, Idaho, Missouri) using propensity score matching (PSM). We then employed DID models to compare changes in two dependent variables, *total payments* and the *number of physicians receiving payments*, across state pairs, controlling for relevant factors.

If the hypothesis holds, we expect to observe varying impacts of the PPSA across individual disclosure states (6). The analysis focused on payments for meals and travel from four pharmaceutical companies (AstraZeneca, Eli Lilly, Novartis, and Pfizer). These firms account for some of the most frequent industry-physician exchanges (13), and have been consistently ranked among the top 10 pharmaceutical companies by sales revenue.^1^,^2^ Meanwhile, meals and travel are two of the top three payment categories in industry–physician financial interactions. For example, according to 2014 Open Payment data, 93.63% of all paid physicians received meals payments, and travel (11.56%) ranked as the third most accepted type of payment by physicians. Thus, our samples demonstrate PPSA's impact on company marketing tactics in the two most frequently used and studied payment categories [e.g., (8, 14)].

We estimated the following DID models:

(1a) $$\log(T\_\mathrm{Pa}y^{j}{)}_{it}=\mu_{i}+\tau_{t}+\beta \mathrm{NoStatePolic}y_{i}\times \mathrm{AfterPPS}A_{t}+\epsilon_{it}$$

(1b) $$\log(P\_\mathrm{Cn}t^{j}{)}_{it}=\mu_{i}+\tau_{t}+\beta \mathrm{NoStatePolic}y_{i}\times \mathrm{AfterPPS}A_{t}+\epsilon_{it}$$

The outcome variables of interest, log(T_Pay*^j^*)*_it_* and log (P_Cnt*^j^*)*_it_*, represent the natural logarithm of the two payment measures, *total payments* and the *number of physicians receiving payments*. $\mathrm{AfterPPS}A_{t}$ is a binary variable indicating whether year *t* occurred after the PPSA implementation. We coded this variable as 1 for 2014–2015 (*after*) and 0 for 2012–2013 (*before*). We used another binary variable, $\mathrm{NoStatePolic}y_{i}$, to indicate whether state *i* had disclosure policy before PPSA implementation. For example, since MN had a prior policy, we set this binary variable to 0, while its matched control state, Idaho, was assigned 1. $\mu_{i}$, the state fixed effects, accounted for any time-invariant factors related to state *i*, such as state population and economic growth; while $\tau_{t}$, the time fixed effects, captured any state-invariant influences related to year *t*, such as market demands. The coefficient for the interaction term $\left(\mathrm{NoStatePolic}y_{i}\times \mathrm{AfterPPS}A_{t}\right)$, β, was the DID estimate that captured the impact of the PPSA on the companies' payment response between the control/disclosure and treatment/non-disclosure states. Our hypothesis would be supported if the significance of β varies across state pairs.

## 3 Results

Table 1 presents DID estimates, confirming that MA's policy led to a significant reduction in industry payments to physicians. MA's success likely stems from its clear requirements, publicly accessible data, and enforcement through penalties for non-compliance. For travel payments, MA's disclosure policy led to a marked 44% decline in the total payments to physicians, estimated by [*e*^(−0.571)^ − 1]. In contrast, ME, MN, and WV's policies had negligible impact, likely due to weaker enforcement, higher reporting thresholds, limited data accessibility, and incomplete coverage of healthcare professionals. For example, MN and WV only required payments higher than $100 to be reported, excluded certain physician categories, and offered little to no public access to raw data.

**Table 1:** DID models (2012–2015) estimate the impact of state disclosure policy^a^^,^^b^.

Control state	Treatment state	Meals		Travel	
		log(T_Pay^M^)	log(P_Cnt^M^)	log(T_Pay^T^)	log(P_Cnt^T^)
MA	MD	−0.027	0.024	−0.571**	−0.283
		(0.071)	(0.044)	(0.064)	(0.067)
		*P* = .74	*P* = .64	*P* = .01	*P* = .05
AIC:		−1.6	−3.5	−1.9	−1.7
ME	OK	0.356	0.325	−0.610	−0.075
		(0.115)	(0.086)	(0.616)	(0.172)
		*P* = .09	*P* = .06	*P* = .43	*P* = .71
AIC:		0.4	−0.7	7.1	2.0
MN	ID	−0.229	−0.079	−0.708	−0.499
		(0.075)	(0.059)	(0.175)	(0.141)
		*P* = .09	*P* = .31	*P* = .06	*P* = .07
AIC:		−1.3	−2.3	2.1	1.2
WV	MO	0.117	−0.026	0.318	0.339
		(0.060)	(0.030)	(0.331)	(0.088)
		*P* *=* *.*19	*P* *=* *.*47	*P* *=* *.*44	*P* *=* *.*06
AIC:		−2.2	−5.0	4.6	−0.7

^a^ T_Pay denotes *Total Payments*, P_Cnt denotes *Number of Physicians Receiving Payment*. Superscript **^M^**denotes meals, and **^T^**denotes travel. **p < .05.

^b^ DID estimator is reported. For significant DID estimators, the average treatment effect is reported as a percentage, calculated as [*e*^(estimated coefficient)^ − 1]*100%. All payments are converted to 2013 U.S. dollars. The DID regression includes year and state fixed effect with robust standard errors reported in parentheses. A negative and significant DID estimator implies that the associated state policy reduces the corresponding payment metric, i.e., either total payments or the number of physicians receiving payments.

AIC (Akaike Information Criterion) is a measure that compares model fit, balancing explanatory power and complexity. Lower AIC values indicate better-fitting models, while penalizing unnecessary parameters.

To further investigate the underlying reason for such varying effects of state disclosure policies, we conducted a comprehensive comparison. Table 2 summarizes differences in reporting thresholds, data accessibility, and enforcement mechanisms across states' disclosure laws. MN, the first to implement a policy in 1994, required only annual, aggregated disclosures covering a narrow scope of payments; the data were not publicly accessible until 2010, and enforcement was minimal. Maine had similar limitations: annual aggregated disclosures, delayed public access until 2010, data published only as PDFs, and minimal enforcement. WV's policy featured vague reporting requirements, limited public access, and virtually no enforcement. VT, although public reported, had restrictions due to trade-secret exemptions and delayed reporting for some payment types.

**Table 2:** Variation in U.S. state and federal disclosure policies^a^.

Policy	Start year(s)	Reporting entities	Covered recipients	Data availability	Disclosed transactions	Enforcement
PPSA (Federal)	2013	Pharmaceutical & Device Manufacturers	Physicians, Teaching Hospitals	Open access; online searchable database; downloadable raw data; aggregated reports	Payments ≥$10; report value, nature, and purpose; categories: general, research, ownership	Civil fines; combined penalties up to $1,150,000
Massachusetts (MA)	2009; Amended 2012	Pharmaceutical & Device Manufacturers	Physicians, Dentists, NPs, PAs, Pharmacists; Facilities: Hospitals, Nursing Homes, Labs, Hospice	Available online; limited searchability; raw data difficult to download	Payments ≥$50; includes CME compensation; ban on gifts; meal ban amended 2012	Fine up to $5,000 per knowing/willful violation
Maine (ME)	2003; Repealed 2011	Pharmaceutical Manufacturers & Drug Labelers	All licensed ME healthcare professionals & entities	PDF with aggregated results only; no raw data	Payments ≥$25; categories: food, travel, samples, gifts	Fine $1,000 plus attorneys’ fees
Minnesota (MN)	1994; Amended 2005, 2012; Repealed 2013	Pharmaceutical Wholesalers & Manufacturers	Physicians, Dentists, Optometrists, Podiatrists; Facilities: Hospitals, Medical Schools	Available only by email request; no online searchability	Payments ≥$100; gifts prohibited >$50; no reporting for 2012	Fine up to $10,000 per violation
West Virginia (WV)	2004; Amended 2006; Repealed 2011	Pharmaceutical Manufacturers & Drug Labelers	Prescribers, Pharmacists, Patient/Consumer Groups	Aggregated reports only; no raw data; no reports after 2010	Payments ≥$100; no individual payment disclosure; no physician IDs	No defined enforcement mechanism
Vermont (VT)	2002; Amended 2009, 2011	Pharmaceutical Manufacturers	Physicians, NPs, PAs, Pharmacists, Health Plan Admins; Facilities: Hospitals, Nursing Homes	Publicly accessible electronic reports; restricted by trade-secret exemptions	Payments ≥$25; disclose rep identity; gift ban (2009); report OTC samples (2011)	Fine up to $10,000 plus attorneys’ fees
District of Columbia (DC)	2004	Pharmaceutical Manufacturers & Drug Labelers	Physicians; general advertising to public	Annual aggregate report only	Payments ≥$25; includes food, travel, samples, education, ads	Fine $1,000 plus attorneys’ fees

HCP, healthcare practitioners; NPs, nurse practitioners; PAs, physician assistants; CME, continuing medical education.

^a^ State policies compiled from state websites, news media, and multiple reports from Policy and Medicine.

By contrast, MA's 2009 policy was comprehensive. It requires pharmaceutical and medical device manufacturers to adopt a detailed code of conduct, including employee training and annual compliance audits. It mandates annual reporting of all payments of at least $50 per transaction, detailing the value, nature, purpose, and specific recipient on an individual, non-aggregated basis, making the data more granular than most peer state laws. Reports are stored in a searchable public database, enhancing accessibility. The law also includes strong enforcement mechanisms, penalizing knowing and willful violations with fines up to $5,000 per transaction and allowing judicial review of penalties, underscoring its credibility and deterrent effect. Together, these features make MA's policy effective in promoting transparency, accountability, and trustworthy industry–physician interactions.

After 2013, payments declined uniformly across all states, suggesting that the PPSA created a policy floor that effectively rendered weaker state laws obsolete. This pattern underscores the need for policymakers to move beyond symbolic gestures and adopt regulations that meaningfully shape corporate behavior. While the PPSA's nationwide framework addresses many of these shortcomings, continuous evaluation and refinement remain essential to fully realize its potential. Importantly, these findings are robust to alternative model specifications and placebo tests.

## 4 Discussion

Our findings suggest that not all disclosure policies are equally effective—policy design and enforcement matter. MA's success likely stems from its clear requirements, publicly accessible data, and enforcement through penalties for non-compliance. In contrast, states with narrower scopes, limited transparency, or weak enforcement mechanisms showed no measurable impact, echoing trends reported in the emerging literature on pre-PPSA state laws, despite having disclosure laws in place. These results highlight the need for policymakers to move beyond symbolic gestures and implement regulations that meaningfully guide corporate behavior and produce tangible changes in physician-industry financial relationships. The PPSA's nationwide framework addresses several structural shortcomings identified in recent studies, but continuous evaluation and refinement are necessary to ensure its long-term effectiveness to fully realize its potential.

Fragmented or weak disclosure policies, such as most U.S. state laws, are often insufficient to influence corporate behavior. Similar inconsistencies appear internationally. In Europe, France's Bertrand Law is among the most comprehensive, yet its data remain not fully standardized, searchable, or downloadable, while other countries have adopted laws with narrower scope, higher thresholds, or weaker enforcement (15). Australia and Japan likewise maintain fragmented approaches that lack centralized, standardized reporting. Collectively, these patterns suggest that transparency policies without uniformity and enforcement often fall short of their intended impact.

Our findings align with recent studies, which show that transparency policies are most effective when strong enforcement is paired with public accessibility. Robust disclosure systems, such as those analyzed by Guo et al. (1, 7) and Chao and Larkin (6), have been linked to measurable changes in prescribing and payment behaviors, whereas other studies indicate that transparency alone often yields limited impact (3, 8). By disaggregating the heterogeneous effects of pre-PPSA state regimes, our study complements this body of work and reinforces calls for policies that move beyond symbolic disclosure toward actionable, well-enforced frameworks.

Drawing on these insights, we suggest that European countries, Australia, and Japan could all benefit from centralized disclosure systems modeled on the PPSA. Such systems would ensure comprehensive reporting across all companies, standardized and publicly accessible data, and strong enforcement mechanisms, including audits and penalties, to promote compliance. Expanding coverage to additional payment types and incorporating periodic evaluation would further enhance transparency for policymakers, researchers, and the public, aligning with best practices observed in the U.S. and France.

This study has some limitations. First, it focuses on only two payment categories (meals and travel) due to data availability. Second, missing or inconsistent data in certain states may limit the generalizability of our findings beyond the included states and companies. Future research integrating broader datasets, additional payment types, and cross-national comparisons informed by post-PPSA literature could provide a more comprehensive understanding of policy effectiveness.

Our findings underscore the critical role of well-designed, effectively enforced policies in achieving meaningful transparency, reinforcing the need for stronger, more uniform federal regulations to mitigate conflicts of interest. Situating these results within the post-PPSA literature demonstrates that both U.S. and international experiences converge on the importance of policy design, enforcement, and standardization.

Building on our findings, together with prior literature, industry reports, and public feedback, several areas for improvement emerge:

**Increase Public Awareness:** Promote the Open Payments database among patients, insurers, and researchers to maximize its utility and influence.

**Strengthen Enforcement:** Implement regular audits and impose penalties for non-compliance to improve data accuracy and deter unethical practices.

**Encourage Innovation**: Support ethical marketing alternatives, such as e-detailing, which maintain transparency while fostering constructive industry-physician collaboration.

## Data Availability

Data sourced from the Open Payments database are publicly available and can be shared. Data obtained from ProPublica's Dollar for Docs database are proprietary and not available for sharing.

## Footnotes

^1^ https://cen.acs.org/articles/94/i10/Biotech-sales-earnings-outpace-big.html

^2^ https://www.pharmaceutical-technology.com/features/featurethe-top-10-biggest-pharmaceutical-companies-of-2014-4396561/
